# Efficacy and Safety of Transcutaneous Electrical Acupoint Stimulation for Postoperative Pain: A Meta-Analysis of Randomized Controlled Trials

**DOI:** 10.1155/2022/7570533

**Published:** 2022-04-14

**Authors:** Dan Wang, Hongshuo Shi, Zhenguo Yang, Wenbin Liu, Lu Qi, Chengda Dong, Guomin Si, Qi Guo

**Affiliations:** ^1^College of Traditional Chinese Medicine, Shandong University of Traditional Chinese Medicine, Jinan, China; ^2^The Second Affiliated Hospital of Shandong University of Traditional Chinese Medicine, Jinan, China; ^3^First Clinical Medical College, Shandong University of Traditional Chinese Medicine, Jinan, China; ^4^Department of Traditional Chinese Medicine, Provincial Hospital Affiliated to Shandong First Medical University, Jinan, China; ^5^Department of Aviation Disease, The 960 Hospital of PLA, Jinan, China

## Abstract

**Objective:**

This meta-analysis aims to evaluate the effectiveness and safety of transcutaneous electrical acupoint stimulation (TEAS) in treating post-operative pain.

**Methods:**

This meta-analysis was registered in PROSPERO (CRD42021286753). We searched PubMed, Embase, and the Cochrane Library for relevant randomized controlled trials (RCTs) about TEAS in treating postoperative pain that were published before November 2021. The primary outcome was visual analogue scale (VAS) within 24 h after surgery. The secondary outcomes included postoperative opioid analgesic drug consumption and the occurrence of adverse reactions within the postoperative 24–72 h. Adverse reactions included dizziness, nausea, and vomiting. Continuous variables were analyzed using mean difference (MDs) or standardized mean difference (SMDs) and 95% CIs. Relative risk (RR) and 95% CI were used for dichotomous data. The data were pooled and analyzed by RevMan 5.4 and STATA15.0 software.

**Results:**

Seventeen trials with 1375 participants were included. The current results suggested that application of TEAS showed obvious superiority in reducing VAS scores (SMD = −1.51, 95% CI = −2.20∼−0.82, I2 = 96%). Subgroup analysis was performed according to open surgery and minimally invasive surgery. VAS scores were decreased after surgery at 24 h (SMD = −0.84, 95% CI = −1.07∼−0.6, I2 = 96%; SMD = −0.88, 95% CI = −1.02∼−0.75, I2 = 96%). The incidence of postoperative dizziness and nausea and vomiting was significantly lower in the TEAS group within postoperative 24–72 h (RR = 0.48, 95% CI = 0.34∼0.68, I2 = 0%; RR = 0.66, 95% CI = 0.44∼1.01, I2 = 69%; and RR = 0.49, 95% CI = 0.24∼1.00, I2 = 51%). Postoperative opioid analgesics were also reduced in the TEAS group within 72 h after surgery (SMD = −2.10, 95% CI = −3.37∼−0.82, I2 = 96%).

**Conclusions:**

TEAS can reduce postoperative pain as well as the incidence of dizziness, nausea, and vomiting and the number of analgesics used after surgery. TEAS is a reasonable modality to incorporate into a multimodal management approach for postoperative pain.

## 1. Introduction

Postoperative pain, including acute postoperative pain and persistent chronic postoperative pain, remains a main clinical problem [1]. In 2020, the current International Association for the Study of Pain (IASP) defined pain as “an unpleasant sensory and emotional experience associated with, or resembling that associated with, actual or potential tissue damages” [2]. A 2011 report from the US National Institutes of Health states that more than 80% of patients suffer postoperative pain, with fewer than 50% receiving adequate pain relief [3]. US surveys from 1993, 2003, and 2012 have shown that postoperative pain is common and remains undertreated, and that the distribution and quality of perceived pain have remained largely unchanged [3]. Evidence suggests that less than half of patients who undergo surgery report adequate postoperative pain relief and about 10% of postoperative pain develops into chronic pain [4]. In addition to impairing the patient's comfort, inadequately controlled pain negatively affects quality of life, function, and functional recovery, increases the risk of postsurgical complications. Postoperative pain not only reduces patients' satisfaction about the healthcare system but also prolongs the length of hospital stay and healthcare costs. Postoperative pain management is still based on the use of traditional opioids such as paracetamol, nonsteroidal anti-inflammatory drugs (NSAIDs), and local anesthetics [5]. A recent retrospective review based on more than 300 000 patients across 380 US hospitals showed that about 95% of surgical patients were treated with opioids [3]. However, opioids have many side-effects that range from bothersome to life-threatening, including nausea, vomiting, constipation, oversedation, somnolence, and respiratory depression [6]. NSAIDs can be responsible for several well-known side effects, comprising upper gastrointestinal bleeding and cardiovascular disease [7]. Local anesthetics are widely used in various fields, but the long-term effects of local anesthetics can lead to adverse conditions, such as inhibition of central and respiratory circulation, and even death of patients [8]. Therefore, it is particularly urgent to find a more efficient way to manage postoperative pain.

Transcutaneous electrical acupoint stimulation (TEAS) is a noninvasive form of electrical acupoint stimulation. Instead of traditional acupuncture intervention, which involves inserting a needle into an acupoint and applying manual stimulation including acupuncture and electroacupuncture, the stimulation on the acupoint is delivered through electricity which is delivered through the surface electrodes [9]. Modern medical research has proved that acupuncture treatment can inhibit the body's pain conduction [10], promote local blood circulation [11], improve the immunity of the patients [12], and enhance the body's anti-inflammatory and metabolic ability [13]. All of these mechanisms can have a rapid analgesic effect. However, there is still a lack of strong clinical evidence to confirm its effectiveness and safety in treating patients with postoperative pain. Therefore, we performed this meta-analysis to assess the effectiveness and safety of TEAS in the treatment of postoperative pain. The primary outcome is the visual analogue scale (VAS) within 24 h after surgery. While the secondary outcomes include postoperative analgesic drug consumption and the occurrence of adverse reactions within postoperative 24–72 h. Adverse reactions included dizziness, nausea, and vomiting.

## 2. Materials and Methods

### 2.1. Compliance with Ethics Guidelines

This article is based on previously conducted studies and does not contain any studies with human participants or animals performed by any of the authors.

### 2.2. Inclusion and Exclusion Criteria

Two authors independently identified the eligibilities of articles for in-depth examination by using the following inclusion: (1) The type of research should be a randomized controlled trial (RCT), and the language is limited to English. (2) Patients of any age and gender with postoperative pain, and if there are other causes of pain will be excluded. (3) The intervention in the experimental group was TEAS (patients in this group received electrical stimulation on the target acupoints. The stimulation was provided by an electrical stimulator through electrode tabs on the target acupoints. The electrical stimulator was set at certain modes, frequency, and intensity accordingly), and the control group was treated with sham-TEAS, blank control, or the same intervention as the treatment group other than TEAS will also be included. (4) Articles involved in evaluating the effectiveness of TEAS on postoperative pain. The exclusion criteria were as follows: (1) article types of comments, case reports, crossover studies, letters, editorials, review articles, meta-analysis, and retrospective studies; (2) studies of animal experiments; and (3) studies involving data that cannot be extracted or lacking adequate data. If discrepancies existed, final decisions were reached via consensus of all authors.

### 2.3. Search Strategy

The meta-analysis was performed in accordance with the Cochrane Handbook for Systematic Reviews of Interventions [14] and is reported in compliance with the PRISMA (Preferred Reporting Items for Systematic Reviews and Meta-Analyses) statement [15]. This meta-analysis was prospectively registered in PROSPERO (CRD42021286753). We searched PubMed, Embase, and the Cochrane Library from inception to November 2021, without any restrictions. The search terms included terms related to TEAS (e.g., “ transcutaneous electrical acupoint stimulation “ OR ” TEAS ” OR “ transcutaneous acupoint electrical stimulation “ OR′ ” acustimulation ”) and terms related to postoperative pain (e.g., ' post-operative analgesi^*∗*^“ OR “ pain, post-operative “ OR “ pain management “ OR′ ” ache^*∗*^”) (Table 1). There were no restrictions on dates, sex, or age, or type of surgery. We searched for these terms in the titles and abstracts of potentially relevant papers. The references of the retrieved papers were also reviewed for further relevant studies. The lists of references of retrieved articles will be searched for identifying potentially eligible trials.

### 2.4. Data Extraction and Outcomes Assessment

All data extraction was independently undertaken by 2 reviewers using predesigned forms. Clinical features (participants, interventions, and outcome measurements), details of the treatments, methodological characteristics, and the results of each outcome were extracted for each study. Discrepancies were handled by discussion. The following information was extracted from each trial: author, year, population, sample size, drug regimen (pathway and dose), and outcome. The primary efficacy outcome was VAS within 24 h after surgery. A VAS score of 0 indicated no pain, and a VAS score of 10 indicated the most severe pain. The secondary efficacy outcomes included postoperative opioid analgesic drug consumption and the occurrence of adverse reactions within postoperative 24–72 h. Adverse reactions included dizziness, nausea, and vomiting.

### 2.5. Quality Assessment and Certainty of Evidence

The Cochrane Collaboration's tool was used to evaluate the risk of bias [16] in the methodology of the included literature. We reviewed each trial and classified the risks as high, low, or unclear, including the following domains: random sequence generation, allocation concealment, blinding of participants and personnel, blinding of outcome assessments, incomplete outcome data, selective reporting, and other biases, such as sample size. Trials rated a high risk of bias in 1 or more areas will be rated high risk, while trials rated a low risk of bias in all aspects will be rated low risk. Two researchers independently performed the quality evaluation of the included articles.

### 2.6. Statistical Analysis

RevMan 5.4 and STATA14.0 software provided by the Cochrane Collaboration were used for data analysis. Continuous variables were analyzed using mean difference (MDs) or standardized mean difference (SMDs) and 95% CIs. Relative risk (RR) and 95%CI were used for dichotomous data. The heterogeneity between the results of the study was examined using the *Q* test (test level is *α* = 0.1), and the magnitude of heterogeneity was judged by combining the findings with the I2 test. Heterogeneity is expressed as *p* and I2; if *p* > 0.10 and I2  <  50%, a fixed effects model was adopted; otherwise, a random effects model was chosen. Sensitivity analysis and subgroup analyses were conducted to assess the stability of results and detect the potential source of heterogeneity. Publication bias was analyzed by performing funnel plots qualitatively and estimated by Egger's test quantitatively.

## 3. Results

### 3.1. Trial Selection

**Figure 1:**
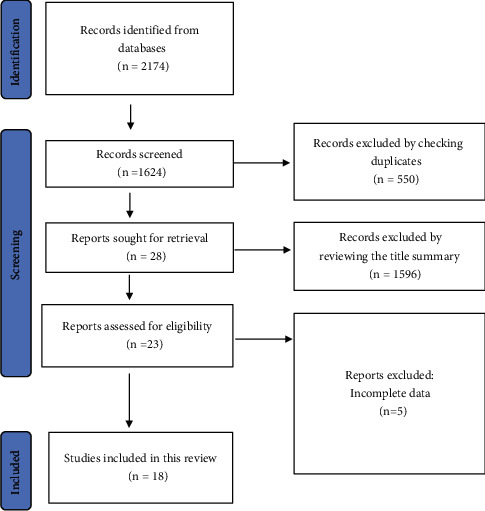
Flow diagram of study selection.

Based on our search strategy, a total of 1277 articles were extracted from the above databases. We first excluded 404 repetitive articles, and then we excluded 852 articles according to the title and abstract. Then, 21 articles were identified for full-text review. Of these, 5 articles were excluded for lack of complete data. Finally, 16 studies [17–32] were included (Figure 1).

### 3.2. Trial Characteristics

**Figure 2:**
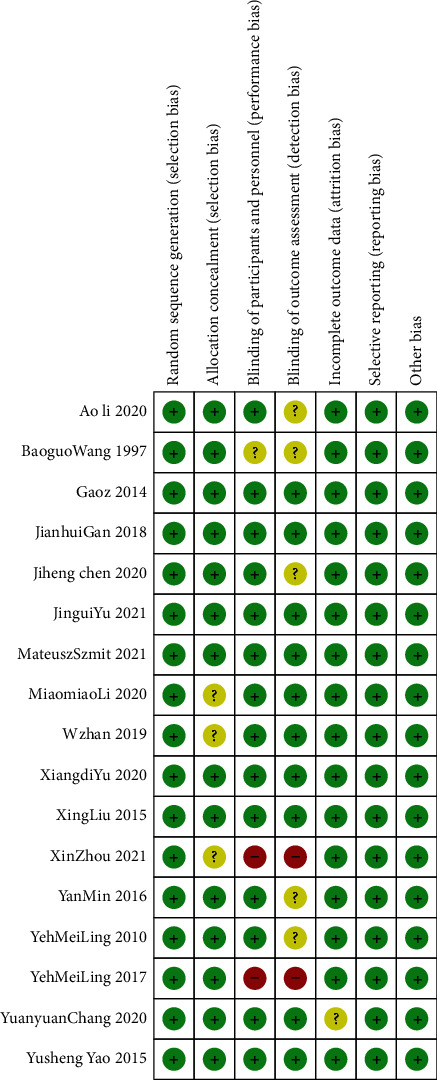
Potential risk of bias of each included study. *Note.* “+” represents low risk; “?” represents unclear risk; and “-” represents high risk.

The characteristics of the included trials are presented in Tables 2 and 3. The trials were published between 1997 and 2021. Among the 16 RCTs included, 11 papers were published in the last 5 years (64.7%). Sample sizes ranged from 24 to 90 patients, and a total of 1305 patients were included, with 651 (49.8%) in the TEAS group and 654 (50.1%) in the control group. The population mainly involved patients with pain after surgery. All trials reported efficacy and safety outcomes. The details of the risk of bias assessment for each included trial are summarized in Figure 2. Overall, 7 trials were classified as low risk of bias, 8 as unclear risk of bias, and 1 as high risk.

### 3.3. Efficacy and Safety of TEAS for the Treatment of Postoperative Pain VAS

**Figure 3:**
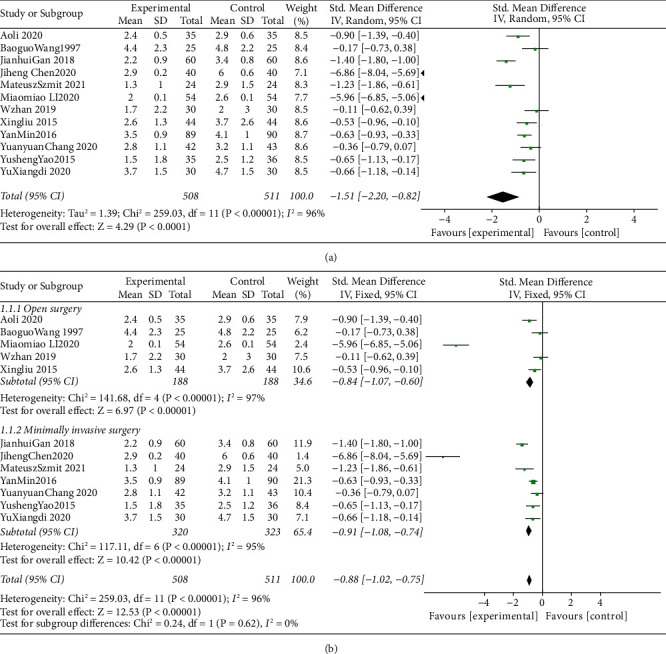
(a) Forest plots comparing the VAS at 24 h between the TEAS and control groups; (b) subgroup analysis of the effect of TEAS for open surgery and minimally invasive surgery.

The meta-analysis combined data from 1019 participants (control group = 511 and intervention = 508). We used a standardized mean difference model to complete a meta-analysis of the pain degree at 24 h after the TEAS intervention in these twelve RCTs. Through meta-analysis, we found that TEAS can significantly reduce VAS scores of patients (SMD = −1.51, 95% CI = −2.20∼−0.82, I2 = 96%) (Figure 3(a)). Then, we stratified the study according to the type of open surgery and minimally invasive surgery (Figure 3(b)). SMD shows that TEAS can significantly reduce the VAS scores when open surgery is performed (SMD = −0.84, 95% CI = −1.07∼−0.6, I2 = 96%). We found that VAS scores also decreased significantly after minimally invasive surgery (SMD = −0.88, 95%CI = −1.02∼−0.75, I2 = 96%).

### 3.4. Incidences of Postoperative Dizziness

**Figure 4:**
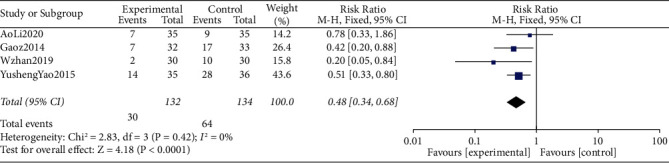
Forest plots comparing the incidence of postoperative dizziness between the TEAS and control groups.

The meta-analysis combined data from 266 participants (control group = 134 and intervention = 132). Four studies compared the incidence of postoperative dizziness within postoperative 24–72 h. The incidence of dizziness was significantly lower in the TEAS group than in the control group (RR = 0.48, 95% CI 0.34∼0.68, I2 = 0%) (Figure 4).

### 3.5. Incidences of Postoperative Nausea

**Figure 5:**
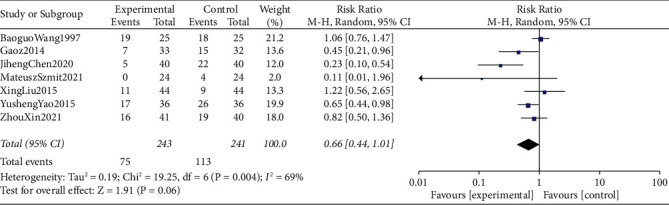
Forest plots comparing the incidence of postoperative nausea between the TEAS and control groups.

The meta-analysis combined data from 484 participants (control group = 241 and intervention = 243). Seven studies compared the incidence of postoperative nausea within postoperative 24–72 h. The incidence of postoperative nausea was lower in the TEAS group than in the control group, but this was not statistically significant (RR = 0.66, 95% CI 0.44∼1.01, I2 = 69%) (Figure 5).

### 3.6. Incidence of Postoperative Vomiting

**Figure 6:**
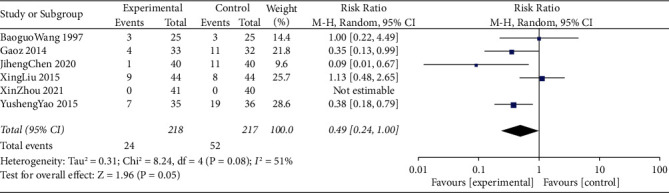
Forest plots comparing the incidence of postoperative vomiting between the TEAS and control groups.

The meta-analysis combined data from 435 participants (control group = 217 and intervention = 218). Six studies compared the incidence of postoperative vomiting within postoperative 24–72 h. Among them, the article XinZhou 2021 did not have the occurrence of vomiting. Compared with the control group, the TEAS group significantly reduced the incidence of postoperative vomiting (RR = 0.49, 95% CI = 0.24∼1.00, I2 = 51%) (Figure 6). We then conducted a sensitivity analysis to further explore the heterogeneity of included studies, which showed that the results of studies were relatively stable and reliable.

### 3.7. Postoperative Opioid Analgesic Consumption

**Figure 7:**
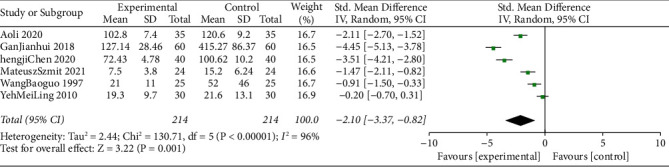
Forest plots comparing the incidence of postoperative analgesic consumption between the TEAS and control groups.

The meta-analysis combined data from 428 participants (control group = 214 and intervention = 214). Six studies compared postoperative opioid analgesic consumption. Compared with the control group, the TEAS group significantly reduced postoperative analgesic consumption (SMD = −2.10, 95% CI = −3.37∼−0.82, I2 = 96%) (Figure 7). The sensitivity analysis findings indicated that the results were robust and reliable.

### 3.8. Publication Bias Analysis and Sensitivity Analysis

**Figure 8:**
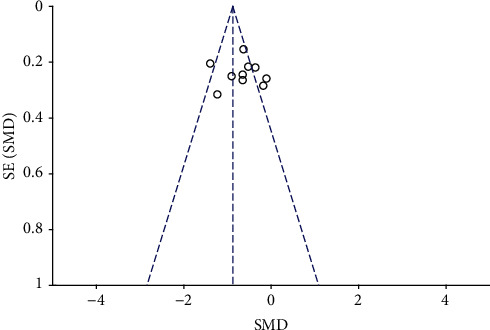
Funnel plot of 24 h VAS score.

There are more than 10 studies on the VAS score after surgery. Sensitivity analyses were performed by removing one study each time to assess the influence of an individual study on the overall outcomes. No significant changes were observed after combining the results, indicating that the results of the study were relatively stable. Then, Egger's test (*p*=0.32) was used to detect publication bias and showed that there was no possibility of publication bias, besides, there was no publication bias by observing the funnel chart (Figure 8).

## 4. Discussion

TEAS has been widely accepted and used worldwide. To our knowledge, our meta-analysis is the first to research the efficacy and safety of TEAS in treating postoperative pain. Our research shows that TEAS can significantly decrease VAS scores of patients. According to subgroup analysis, we found that VAS scores decreased significantly after minimally invasive surgery and open surgery. Minimally invasive surgery is the direction of surgery [30]. TEAS selections need to be considered to improve the efficacy and clinical quality of patients. In addition, our study revealed that TEAS provides broadly generalizable benefits during the postoperative recovery period and helps to accelerate the progress of enhanced recovery after surgery.

According to the theory of traditional Chinese medicine, acupuncture meridians represent “channels” through which energy called “meridian qi” flows [31]. Acupuncture has been utilized in Chinese health care for at least 2500 years, which is a technique for balancing the flow of energy [32]. The underlying mechanisms of TEAS's analgesic effects have not been clearly clarified. Basic studies have shown that TEAS can achieve the intervention effect on pain sensation and can be exerted via multiple mechanisms. (1) TEAS may produce analgesia by promoting the release of endogenous opioid peptides [33]. (2) TEAS inhibit the production of endogenous pain-causing substances [33]. (3) TEAS to intervene in the MAPK signal transduction pathway to play analgesic effect [34]. (4) TEAS inhibit pain sensitization. The early peripheral sensitization of neuropathic pain may be interfered by downregulating TRPV1 phosphorylation level and calcitonin gene-related peptide expression level of injured DRG [35].

Postoperative pain contributes to increased morbidity, impaired physical function and quality of life, slowed recovery, and increased cost of care [36]. Given the unclear formation mechanism of postoperative pain, it remains one of the most challenging problems in clinical pain therapeutics. Studies have shown that TEAS could regulate the function of the hypothalamic pituitary-adrenal (HPA) axis and antagonize the hyperfunction of the HPA axis [37]. The HPA axis has many functions including regulation of appetite, sleep, sexual desires, and adaptation to stress [38]. Dysfunction of the HPA axis is thought to be primarily responsible for psychological/behavioral symptoms (pain sensitivity, depression, and fatigue) [38]. An RCT showed that TAES treatment can increase the serum levels of IL-2 and IFN-*γ*, and decreased IL-4 secretion and return the aforementioned cellular immune factors to the preoperative control value at a faster rate [39]. These results suggest that TAES can reduce postoperative immune dysfunction by changing the expression of Th1/Th2 cell-associated cytokines [39]. Simultaneously, some preclinical studies have also shown that TEAS can attenuate cognitive deficits by inhibiting neuronal peroxide reactions in hippocampus tissue and inflammation of the central and peripheral nervous systems [40]. The major pathway is the cholinergic anti-inflammatory pathway (CAP). TEAS can stimulate the vagus nerve to activate CAP so as to inhibit the production of proinflammatory cytokines [41]. Studies have revealed that low-frequency electrical stimulation can release enkephalins and endorphins from the central nervous system [42]. High frequency electrical stimulation induces the release of endorphins from the spinal cord. Low frequency/high frequency alternating density waves can simultaneously stimulate these three peptides to produce a synergistic analgesic effect [43]. TEAS may affect 5-HT transmission by activating 5-HT and norepinephrine fibers to promote gastrointestinal motility and reduce the incidence of nausea and vomiting [44]. Meanwhile, there is a dose-response relationship between opioid dosage and associated side effects [45]. Opioid analgesics are commonly used postoperative analgesics in clinics, but they easily cause dose-dependent respiratory depression, gastrointestinal reaction, urinary retention, skin itching, and other adverse reactions [46]. Our study revealed that the application of TEAS was associated with lower opioid analgesic consumption. Therefore, TEAS provides a nondrug alternative for multimodal analgesia for postoperative pain.

## 5. Conclusions

TEAS is a reasonable modality to incorporate into a multimodal management approach for postoperative pain. TEAS can reduce postoperative pain as well as the incidence of dizziness, nausea, and vomiting and the number of analgesics used after surgery. Owing to the limitations, further large-scale and well-designed studies are required to verify and expand on our conclusion.

## Figures and Tables

**Table 1:** Embase search terms.

Number	Search terms
^#^1	“Postoperative pain”/exp
^#^2	“Postoperative pain”:ab, ti
^#^3	“Postoperative analgesi^*∗*^”:ab, ti
^#^4	“Pain management”:ab, ti
^#^5	ache^*∗*^:ab, ti
^#^6	^#^1 OR ^#^2 OR ^#^3 OR ^#^4 OR ^#^5
^#^7	“Transcutaneous electrical acupoint stimulation”:ab, ti
^#^8	“Transcutaneous acupoint electrical stimulation”:ab, ti
^#^9	electroacupuncture^*∗*^:ab, ti
^#^10	“Electro acupuncture”:ab, ti
^#^11	teas: ab, ti
^#^12	^#^7 OR ^#^8 OR ^#^9 OR ^#^10 OR ^#^11
^#^13	^#^6 AND ^#^12

**Table 2:** Characteristic of the included studies.

Trial	Year	Sample size		Interventions		Acupoint selections	Target outcomes	Type of surgery
		TEAS group	Control group	TEAS group	Control group			
AoLi [14]	2020	35	35	TEAS	Shame teas	Hegu (LI4),Neiguan (PC6), Zusanli (ST36)	5/3/1	Breast cancer surgery
JihengChen [15]	2020	40	40	TEAS	Shame teas	Hegu (LI4), Neiguan (PC6), Houxi (SI3), Zhigou (TE6)	5/3/4	Lung cancer surgery
MiaoMiaoLi [16]	2020	54	54	TEAS	Shame teas	Zusanli (ST36)	2/3	Cesarean section
XingLiu [17]	2015	44	44	TEAS	Shame teas	Hegu (LI4), Waiguan (TE5), Jinmen (BL63), Taichong (LR3), Zusanli (ST36), Qiuxu (GB40), Fengchi (GB20), Tianzhu (BL10), Cuanzhu (BL2), Yuyao (EXHN4)	2/3/4	Infratentorial craniotomy
YuanyuanChang [18]	2020	42	43	TEAS	Shame teas	Shenmen (HT7), Neiguan (PC6), Zusanli (ST36), Hegu (LI4)	3	Thoracoscopic surgery
YanMin [19]	2016	89	90	TEAS	Shame teas	Hegu (LI4), Neiguan (P6)	3	Laparoscopic surgery
MateuszSzmit [20]	2021	24	24	TEAS + PCA	Shame teas + PCA	Hegu (LI4), ashi points	5/2/3	Laparoscopic inguinal hernia surgery
JianhuiGan [21]	2018	60	60	TEAS	Conventional treatment	Shenyu (BL23), Yinlingquan (SP9)	5/3	Ureteroscopic surgery
BaoguoWang [22]	1997	25	25	TEAS + PCA	Shame teas + PCA	Hegu (LI4)	5/2/3/4	Lower abdominal surgery
YushengYao [23]	2015	35	36	TEAS	Shame teas	Hegu (LI4), Neiguan (PC6), Zusanli (ST36), Sanyinjiao (SP6)	2/3/1/4	Gynecological Laparoscopic surgery
YehMeiLing [24]	2010	30	30	TEAS	Shame teas	Weizhong (BL40), Yanglingquan (GB34), Shenmen (HT7), Neiguan (P6)	5	Gynecological Laparoscopic surgery
YehMeiLing [25]	2017	39	41	TEAS	Conventional treatment	Chengshan (BL57), Erbai (EX-UE2)	3	Hemorrhoid resection
XiangdiYu [26]	2020	30	30	TEAS	Shame teas	Baihui(GV20), Yingtang(EX-HN3), Zusanli (ST36), Neiguan (PC6)	3	
Gaoz [27]	2014	33	32	TEAS	Shame teas	Hegu (LI4), Neiguan (PC6), Zusanli (ST36)	2/1/4	Ambulatory breast surgery
XinZhou [28]	2021	41	40	TEAS	Conventional treatment	Hegu (LI4), Neiguan (PC6), Weishu (BL21), Xiaochangshu (BL27), Zusanli (ST36), Shangjuxu (ST37)	2/4	Laparoscopic surgery for gastric cancer
Wzhan [29]	2019	30	30	TEAS + TAP	TAP	Zusanli (ST36), Neiguan (PC6)	3/1	Abdominal surgery

*Note.* TEAS, transcutaneous electrical acupoint stimulation; VAS, visual analogue scale; 1. postoperative dizzy; 2. postoperative nausea; 3.VAS; 4. postoperative vomiting; 5. postoperative analgesic dosage.

**Table 3:** Details of interventions.

Trial	Year	Time point	Postoperative opioid analgesics
AoLi [14]	2020	30 min before induction of anesthesia at 4 and 12 h postoperation	PCA:150 ml Sufentanil 1.5 *μ*g/kg if needed
JihengChen [15]	2020	30 min before induction, throughout the surgical, and 6, 24, and 48 h; sufentanil 1.5 *μ*g/kg if needed postoperation	PCIA : sufentanil 1.5 *μ*g/mL if needed
MiaoMiaoLi [16]	2020	60 min postoperative and twice times on the next 24, 48, and 72 h after surgery	Not mentioned
XingLiu [17]	2015	30 min before induction	Not mentioned
YuanyuanChang [18]	2020	30 min preoperative, the end of surgery and 24 and 48 h after surgery	Not mentioned
YanMin [19]	2016	30 min before induction	Not mentioned
MateuszSzmit [20]	2021	30 min at intervals of 2 h within 24 hours after surgery	PCA: morphine 15 ml if needed
JianhuiGan [21]	2018	30 min at 4, 8, and 12 h postoperatively and three times on the next 2 days after surgery	Tramadol hydrochloride tablets if needed
Baoguo Wang [22]	1997	30 min every 2 h on the next 2 days after surgery	PCA: hydromorphone 1 or 2 mL if needed
YushengYao [23]	2015	30 min before the induction of anesthesia	Not mentioned
YehMeiLing [24]	2010	20 min at 2 and 4 h after surgery	PCA: morphine 1 mg if needed
YehMeiLing [25]	2017	20 min at 4, 6 h and at 7 a.m. and 11 a.m. on the next day after surgery, 4 times in total	Not mentioned
XiangdiYu [26]	2020	30 min before induction	Not mentioned
Gaoz [27]	2014	30 min before induction	Not mentioned
XinZhou [28]	2021	30 min at 8 : 00 a.m. and 4 : 00 p.m. on the next 3 days after surgery	Not mentioned
Wzhan [29]	2019	30 min preoperative and postoperative	Not mentioned

*Note.* TEAS, transcutaneous electrical acupoint stimulation.

## Data Availability

The datasets analyzed during the current study are available from the corresponding author on reasonable request.
